# Probabilistic Sampling Networks for Hybrid Structure Planning in Semi-Structured Environments

**DOI:** 10.3390/s25206476

**Published:** 2025-10-20

**Authors:** Xiancheng Ji, Jianjun Yi, Lin Su

**Affiliations:** School of Mechanical and Power Engineering, East China University of Science and Technology, Shanghai 200237, China

**Keywords:** hybrid structure, Dempster–Shafer, motion planning module, fusion sampling module

## Abstract

**Highlights:**

**What are the main findings?**

- A hybrid structure planning method based on a Probabilistic Sampling Network (PSNet) and an Enhanced Artificial Potential Field (EAPF) is proposed to address high-dimensional robot motion planning in semi-structured environments.
- Experiments demonstrate that the proposed method outperforms MPNet and RRT-Connect in both 2-D point-mass robot and 6-DOF manipulator tasks, achieving higher success rates and more stable collision avoidance.

**What is the implication of the main finding?**

- The proposed approach enhances the adaptability and robustness of industrial robots in intelligent manufacturing, maintaining efficient path planning in dynamic and complex scenarios.
- This study provides a new perspective for integrating learning-based methods with classical planning techniques, laying the foundation for future applications in autonomous robotic operations and human–robot collaboration.

**Abstract:**

The advancement of adaptable industrial robots in intelligent manufacturing is hindered by the inefficiency of traditional motion planning methods in high-dimensional spaces. Therefore, a Dempster–Shafer evidence theory-based hybrid motion planner is proposed, in which a probabilistic sampling network (PSNet) and an enhanced artificial potential field (EAPF) cooperate with each other to improve the planning performance. The PSNet architecture comprises two modules: a motion planning module (MPM) and a fusion sampling module (FSM). The MPM utilizes sensor data alongside the robot’s current and target configurations to recursively generate diverse multimodal distributions of the next configuration. Based on the distribution information, the FSM was used as a decision-maker to ultimately generate globally connectable paths. Moreover, the FSM is equipped to correct collision path points caused by network inaccuracies through Gaussian resampling. Simultaneously, an augmented artificial potential field with a dynamic rotational field is deployed to repair local paths when worst-case collision scenarios occur. This collaborative strategy harmoniously unites the complementary strengths of both components, thereby enhancing the overall resilience and adaptability of the motion planning system. Experiments were conducted in various environments. The results demonstrate that the proposed method can quickly find directly connectable paths in diverse environments while reliably avoiding sudden obstacles.

## 1. Introduction

The increasing complexity of intelligent manufacturing systems demands more agile and adaptable robots [1,2]. However, the current mainstay, the offline robot demonstration method, poses limitations. Despite its efficacy in demonstrating intricate trajectories, this method requires specialized expertise and struggles with environmental changes [3]. Any alterations to the working environment or robot necessitate a re-demonstration of the data, hindering the robot’s adaptability. Integrating advanced motion planning algorithms into industrial robots emerges as a critical strategy to address the evolving demands of intelligent manufacturing. Enhancing the robots’ capabilities and flexibility reduces the reliance on specialized expertise and enables seamless adaptation to dynamic workspaces, ultimately facilitating the rapid improvement required in modern manufacturing environments.

Decades of research and development have yielded numerous remarkable contributions to motion planning algorithms. Sampling-based motion planning methods (SBMPMs), such as the rapidly exploring random tree (RRT) [4] and its variants [5,6,7], have successfully solved high-dimensional motion planning problems by sampling in the configuration space. However, SBMPMs compute and optimize collision-free trajectories from scratch each time, which is time-consuming and leads to uncertainty in the planning results. Industrial robots commonly work on several production lines, but each environment is relatively fixed, with potential temporary obstacles being added [8]. In such a semi-structured setting, discarding previous planning data and planning from scratch each time is not always a sensible approach [9].

In effect, extensive data accumulated from previous planning, which contains information about similar situations and their respective near-optimal trajectories, can be translated into previous experience to speed up the computation of near-optimal collision-free trajectories. But this valuable resource is scarcely exploited in SBMPMs. In recent years, the evolution of motion planning and artificial intelligence has crossed, leading to the emergence of learning-based methods [10,11]. These approaches leverage neural networks’ powerful nonlinear mapping capabilities to accelerate motion planning by learning from extensive datasets [12,13]. Despite this, neural network-based planners face a significant challenge: the network mapping accuracy problem. This issue arises from the network’s inherent limitations in directly mapping high-quality trajectories due to limited precision, causing local collisions and oscillations near the target point, potentially leading to the failure of the entire planning path. Some studies alter the mapping result from a trajectory to a distribution, reducing the requirement for network mapping accuracy [14,15]. Nevertheless, these distributions usually still need to be processed by SBMPMs further. Alternatively, making recursive calls to the neural network can enhance planning accuracy, but this approach still cannot prevent all unforeseen circumstances [16]. Furthermore, the network planner rarely escapes collisions when mapped paths result in collisions, demonstrating a weak ability to independently resolve such issues [17].

This paper proposes a Dempster–Shafer-based hybrid structure planner using a probabilistic sampling network (PSNet) and cooperation with an enhanced artificial potential field method (PSNet-EAPF) to boost the effectiveness of robot motion planning. In the proposed method, a motion planning module (MPM) consisting of a multilayer regression network and an encoder is constructed to break the centralized planning task into multiple sub-distributions in each step, which obeys the Gaussian distribution. Based on the sub-distribution information obtained, the fusion sampling module (FSM) in PSNet is employed to fuse and resample them, producing a globally connectable path to mitigate the network mapping accuracy problem. Additionally, the variable rotational field enhances the artificial potential field and cooperates with PSNet to repair the local path and boost the ability to escape collisions if worst-case scenarios occur in the global path. The experimental investigation demonstrated that the proposed method’s performance is satisfactory and superior to that of other similar planning methods.

The primary contributions of the novel probabilistic sampling network integrated with the enhanced artificial potential field method are summarized as follows:

1. Experience-driven probabilistic planning: PSNet integrates prior planning experience into probabilistic sampling, accelerating the computation of feasible trajectories and reducing planning time from minutes to seconds.
2. Robust fusion and Gaussian resampling: A novel fusion sampling module combined with Gaussian resampling corrects local collision errors caused by network inaccuracies, substantially improving success rates and stability.
3. Adaptive hybrid structure: By coupling the learning-based global planner with an enhanced artificial potential field (EAPF), the method achieves real-time local replanning, overcoming the local minima and goal non-reachability problems of traditional potential fields.

The remainder of this paper is organized as follows. A thorough literature review of related work is introduced in Section 2. PSNet and EAPF are described in Section 3. The experimental results are presented in Section 4. The discussions based on the experimental results are included in Section 5. Finally, the conclusions of our work are drawn in Section 6.

## 2. Related Work

SBMPMs were initially introduced to address the resolution completeness problem, which necessitates complete obstacle geometry, a requirement often difficult to fulfill in real-world environments [18,19,20]. However, in practice, SBMPMs demonstrated their ability to efficiently solve motion planning problems in dynamic environments, making them a promising approach. These methods rely on random sampling and collision-checking techniques to find valid collision-free configuration spaces. Connectable paths can then be efficiently discovered by searching the sampled configuration space. The most classic SBMPMs are RRT and probabilistic roadmaps (PRM) [21], which model an exploring tree structure and a roadmap in the collision-free space, respectively. In practice, RRT is preferred over PRM because it does not require the generation of precompiled roadmaps, which can often be difficult to compute quickly in online planning problems. Although RRT performs substantially better than PRM, there is still room for improvement, leading to the development of various RRT variants to enhance their capabilities. RRT-connect, an improved algorithm based on RRT, generates two rapidly exploring random trees from the initial and target configurations simultaneously, making it more efficient than RRT [22]. Wang et al. further improved RRT-connect’s execution efficiency by adding a new sampling node [23]. Chen et al. provided a similar sampling strategy and introduced a guidance module that biases RRT-connect towards the target point during expansion [24]. While RRT can find a path, it does not always find the shortest one. The exploring Random Tree Star (RRT*) algorithm was proposed to overcome this limitation, which is asymptotically optimal [25]. RRT* finds an initial path using RRT and then continuously optimizes it as more samples are added. However, RRT* becomes computationally inefficient as the dimensionality of the planning problem increases. Even with enhanced methods like RRT*-SMART [26], PF-RRT* [27], and PQ-RRT* [28], these approaches still struggle to efficiently solve high-dimensional manipulation problems due to a lack of prior knowledge of the environment.

Recently, researchers have focused on leveraging previous experiences to accelerate new planning tasks instead of starting from scratch [15,29]. This paradigm shift aims to capitalize on the valuable data accumulated from prior planning scenarios and their corresponding near-optimal trajectories, enabling more efficient computation of new collision-free paths. Some of the work has focused on modifying traditional SBMPMs. Fisher R et al. combined Dynamic Reachability Maps (DRM) with RRT-connect. It leverages the topological features of previous paths by using a Reeb graph to capture the persistent characteristics of past solutions, thereby improving efficiency in repetitive tasks [30]. Ref. [31] proposed an Experience-Based Bidirectional RRT algorithm. The algorithm significantly improves the efficiency and quality of path planning by learning a Gaussian Mixture Model (GMM) from demonstration paths to create an adaptive sampler and using an Experience Graph to capture exploration information in the configuration space. Improvements based on similar motivations have also appeared in the RPM algorithm [32,33,34]. This work has greatly enhanced the practicality of SBMPMs in semi-structured environments, but it remains significantly limited because changes in high-dimensional obstacles are difficult to describe within the method. Additionally, the inherent nature of random sampling in these methods can lead to jerky or redundant motions.

Another aspect of the research emphasizes the use of artificial intelligence techniques to deal with the challenges of high-dimensional obstacle representation and leverage prior planning experiences. Ichter et al. used RRT to sample paths in learned latent spaces [35], but this method cannot ensure that path solutions exist in the learned latent space. In [36], a CNN network learned from images to guide sampling. Ref. [37] employed 3D point cloud reconstruction from image sets by a network and an enhanced MSRRT* algorithm to efficiently determine paths. Other similar studies [38,39] aim to interpret and encode visual information via networks. This method circumvents the manual interpretation of obstacles in high-dimensional joint spaces and offers an essential condition for future motion planning. Jetchev et al. described a method for accelerating planning through experience data, where multiple target trajectories were mapped directly from the trained neural network and selected and optimized by an objective function [14]. This method achieves the expected acceleration effect, but mapping high-quality trajectories directly from the network remains challenging, thereby reducing the efficiency of the subsequent path optimization process. To address this, ref. [28] introduced encoders to switch the mapping result from a trajectory to a distribution, reducing the requirement for network output accuracy. The trajectory was then generated by sampling over the distribution using SBMPMs such as FMT∗ [40] and RRT∗. In addition, reference [13] introduced a Long Short-Term Memory (LSTM) network as the planning module, which recursively invokes planning based on the start and goal positions as retrieval conditions, thereby significantly improving the overall path quality. Meanwhile, Li et al. [41] proposed a modular path planning framework inspired by natural behaviors, enabling adaptive switching among the most advantageous algorithms under varying planning scenarios. Qureshi et al. [17] highlighted the benefits of the recursive call generation trajectory approach and proposed a comprehensive motion planning network model (MPNet). This model consists of environmental information processing and motion planning modules (MPnet-Pnet), incorporating environment information, the robot’s initial and desired goal configurations, and recursively calling motion planning modules to generate connectable paths. If the planned path encounters collisions, the method activates the network’s drop layer (MPNet-Drop) or calls SBMPM to find a new local path. In [42], constraint manifolds were added to MPNet to expand its functionality further. Ying et al. [43] applied a similar planning network model to dual-arm assembly robots, achieving the desired results. Li et al. [44] fused the model-predictive control into MPNet (MPC-MPNet), which introduces two models based on neural generators, discriminators, and parallelizable model predictive controllers to improve MPNet’s planning success rate.

MPNet and its variants have addressed some of the mapping accuracy challenges in network planners, but they have not fully resolved the issue. Moreover, when the mapped target path collides, MPNet struggles to escape the collision region using its built-in re-planning method. The work on MPNet has been a great source of inspiration for us, but our proposed method is distinct from previous approaches as it combines a probabilistic sampling network with a Gaussian sampling module. This module is designed to absorb network errors and enhance the network’s ability to navigate around obstacles. Furthermore, we replace the reliance on SBMPMs for local path generation with a novel artificial potential field enhanced by a variable rotational field.

## 3. Methods

### 3.1. Problem and Notations Definition

In this study, a multi-joint robot configuration space is denoted as $C\subset R^{d}$, where d is the dimension of R. The obstacle space $C_{obs}$ and the obstacle-free space $C_{free}$ are complementary sets ($C_{free}=C-C_{obs}$) of configuration space. Let $k=\{c_{init},c_{2},\ldots ,c_{T}\}$ be the ordered configurations list of the robot’s configurations, where T is the list length, and $\{c_{init},c_{2},\ldots ,c_{T}\}\in C_{free}$. The $k_{i}$ is assumed to be the i th configuration list in one training set, where i∈[1,n], and n is the number of configuration lists. $k_{ij}$ corresponds to the j th robot state ($c_{j}\in [1,T$]) in the i th configuration list. Then, a robot motion planning problem in our study can be denoted as $\left\{c_{init},c_{T},C_{obs}\right\}\to k=\{c_{init},c_{2},\ldots ,c_{T}\}$, which can be interpreted as finding a feasible path solution in $C_{free\;}$connecting the $c_{init}$ to the $c_{T}\;$by a motion planning method. In addition, building a collision checker into the algorithm is necessary. Due to the high dimensionality of the configuration space, which generally depends on the degree of freedom of the multi-joint robot, it is complicated to describe the obstacles in the configuration space. As a result, collision checkers are usually built in a Cartesian space, up to three dimensions, rather than a configuration space. The Cartesian space around the multi-joint robot, also called the workspace, is denoted as $O\subset R^{m}$, where m is a Cartesian space dimension. In the workspace, the obstacle space is denoted as $O_{obs}\in O,$ and the obstacle-free space is denoted as $O_{free}=O-O_{obs}$. The collision checker is denoted as $\Phi \left(k_{ij},k_{ij+1,}O_{obs}\right)\to \{valid,collision\}$, which takes robot configuration information $k_{ij}$, $k_{ij+1,}$ and workspace information $O_{obs}$, and estimates if they are valid or not. The planned trajectory refers to the joint motion trajectory of the robot in configuration space, where each configuration encodes the complete pose of all robot joints. The resulting trajectory thus represents the robot’s full-body motion rather than solely the end-effector’s Cartesian path.

### 3.2. Probabilistic Sampling Network

**Figure 1:**
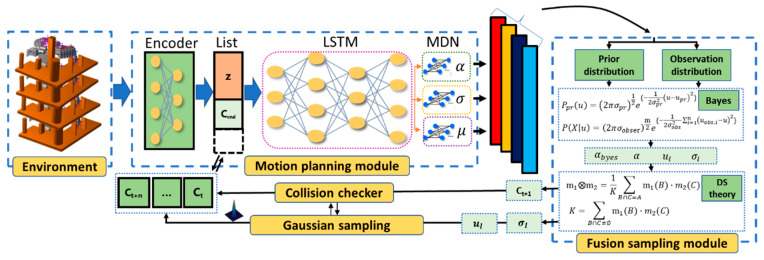
The probabilistic sampling network (PSNet).

The global collision-free path is generated from the start state to the goal state by recursively calling the neural planner PSNet. The PSNet comprises two modules: the MPM and the FSM with Gaussian resampling capability. The MPM takes the environmental information, such as multiple views or point clouds from sensors, the robot’s current configuration, and the goal state to output multimodal distributions of the next configuration. Based on this distribution information, the FSM acts as an information arbiter to produce a globally connectable path. The components of PSNet are shown in Figure 1.

#### 3.2.1. Motion Planning Module:

The motion planning module is a multilayer regression network with an encoder. The environment information $O_{obs}$ is encoded into the latent space Z through the encoder, which can be concisely denoted as:

(1) $$z=Enc\left(O_{obs}\right)$$

Subsequently, the latent space Z, the robot’s initial configurations $c_{init}$ and the target configurations $c_{T}$ are concatenated into a new input list $\left\{c_{init},c_{T},z\right\}$ for the remaining network structure. This structure, akin to the Mixture Density Network (MDN) [45], consists of an LSTM stacking layer (LSTMS) and three independent fully connected layers (IMDN). The LSTMS captures the probability distribution features between the current and next robot states. The IMDN is employed to map the mean $\left[u_{1},\ldots ,u_{m}\right]$, standard deviation $\left[\sigma_{1},\ldots ,\sigma_{m}\right]$ and weight coefficient $[\alpha_{1},\ldots ,\alpha_{m}],$ which are multimodal distribution models $g{(c}_{j+1}|\left\{c_{init},c_{T},z\right\})$ of the next robot configuration, based on the input data.

At this time, the robot motion planning problem $\left\{c_{init},c_{T},C_{obs}\right\}\to k=\{c_{init},c_{2},\ldots ,c_{T}\}$ is transformed into the $\left\{c_{init},c_{T},C_{obs}\right\}\to k=P\left(c_{next}|\left\{c_{init},c_{T},C_{obs}\right\}\right)$ of $c_{next}$, which is equivalent to $P(c_{j+1}|c_{j})$:

(2) $$P(c_{j+1}|c_{j})=\sum_{l}^{m}\alpha_{l}(c_{j})g_{l}(c_{j+1}|\left\{c_{j},c_{T},z\right\})$$

where m is the number of multimodal distribution models and $\alpha_{l}$ is the learned weight coefficient of each model. The $g_{l}(c_{j+1}|\left\{c_{j},c_{T},z\right\})$ represents l-th multimodal distribution model of the form:

(3) $$g_{l}\left(c_{j+1}|\left\{c_{j},c_{T},z\right\}\right)=\frac{1}{\sqrt{2\pi }\sigma_{l}\left(c_{j}\right)}\exp\{-\frac{{\left\|c_{j+1}-u_{l}(c_{j})\right\|}^{2}}{2{\sigma_{l}\left(c_{j}\right)}^{2}}\}$$

where $u_{l}$ and $\sigma_{l}$ both learned from the multilayer regression network like $\alpha_{l}$, denoting mean and standard deviation, respectively. Then, the loss function of multilayer regression network training is defined as the negative log-likelihood:

(4) $$Loss=-ln\{\sum_{l}^{m}\alpha_{l}(c_{j})g_{l}(c_{j+1}|\left\{c_{j},c_{T},z\right\})\}$$

The above process can be simplified as follows:

(5) $$P(c_{j+1}|c_{j})\leftarrow PSNet(\left\{c_{j},c_{T},Enc\left(O_{obs}\right)\right\})$$

#### 3.2.2. Fusion Sampling Module

The classical MDN employs multinomial distribution sampling, a statistical method, to select one distribution and determine the next point. However, this stochastic sampling approach overlooks inherent uncertainty: the selected probability distribution inadequately represents the quality of all sampling points. To address this limitation, our study utilizes Dempster–Shafer (DS) theory [46], recognized for its effectiveness in handling uncertainty, as the core algorithm in our module to replace multinomial distribution sampling, thereby enhancing the robustness of the sampling method.

Firstly, Bayesian estimation theory (BET) is employed to augment the sample. The multimodal distributions $P(c_{j+1}|c_{j})$ are converted into the prior distribution parameters $\sigma_{pr},u_{pr}$ and observation distribution parameters $\sigma_{obser},u_{obser}$ of the BET using a weighted or averaging technique.

(6) $$\sigma_{obser}=\frac{\sum_{l=1}^{m}\sigma_{l}}{m}\;u_{obser}=\frac{\sum_{l}^{m}u_{l}}{m}\;\sigma_{pr}=\sum_{l}^{m}\sigma_{l}\alpha_{l}\;u_{pr}=\sum_{l}^{m}u_{l}\alpha_{l}$$

Then, the BET-based distribution model $P_{byes}(c_{j+1}|c_{j})$ is further calculated:

(7) $$P_{byes}\left(c_{j+1}|c_{j}\right)=(2\pi \sigma_{obser}{)}^{\frac{m}{2}}exp\{-\frac{1}{2\sigma_{0}^{2}}\sum_{i=1}^{m}\left(x_{i}^{obser}-{\bar{x}}^{pr}\right)\}$$

where $x_{i}^{obser}$ is from the $N(u_{obser}{{,\sigma }_{obser}}^{2})$, and ${\bar{x}}^{pr}$ is from the $N(u_{pr}{{,\sigma }_{pr}}^{2})$. When ${\bar{x}}^{pr}$ is replaced in Equation (6) in the form of distribution, and the mean ${\hat{u}}_{byes}$ and standard deviation ${\hat{\sigma }}_{byes}$ of Bayesian estimation. BET fuses these two sources, yielding:

(8) u^byes=mσobser2mσobser2+1σpr2uobser+mσpr2mσpr2+1σobser2x¯pr

This produces a fused Gaussian belief $N({\hat{u}}_{byes},{{\hat{\sigma }}_{byes}}^{2})$, encapsulating both model confidence and observation uncertainty.

Next, we construct the basic probability assignment (BPA) functions of DS theory. In DS theory, the BPA or evidence $m:2^{\Theta }\to \left[0,1\right]$ quantifies the degree of belief assigned to each subset of the frame of discernment. Here, two types of BPA functions are defined:

MDN-derived evidence ($m_{1})$: The first BPA directly maps the original mixture weights to belief assignments.

(9) $$m_{1}\left(\left\{D_{l}\right\}\right)=\alpha_{l},\forall l\in \{1,2,\ldots ,m\}\;\;m_{1}\left(A\right)=0,\forall A\in 2^{\Theta }\setminus \{\{D_{1}\},\{D_{2}\},\ldots ,\{D_{m}\}\}$$

where A represents any subset of the frame of discernment Θ (i.e., any element of the power set $2^{\Theta }$) except the singleton sets $\left\{D_{i}\right\}$. This means we assign zero mass to all compound hypotheses, focusing belief only on individual distributions.

BET-derived evidence ($m_{2})$: The Bayesian estimation is used to derive a Gaussian belief, which is used to assess the likelihood of each Gaussian component.

(10) $$\alpha_{l}^{\mathrm{byes}}=\frac{1}{\sqrt{2\pi }{\hat{\sigma }}_{byes}}\exp\left(-\frac{{\left(\mu_{l}-{\hat{u}}_{byes}\right)}^{2}}{2{{\hat{\sigma }}_{byes}}^{2}}\right)$$

This is normalized across all l, yielding a set of Bayesian-informed weights:

(11) $$\alpha_{byes}(l)=\frac{\alpha_{l}^{\mathrm{byes}}}{\sum_{i=1}^{m}\alpha_{i}^{\mathrm{byes}}}$$

Then, the second BPA $m_{2}$ is derived from the Bayesian estimation.

(12) $$\begin{array}{l} m_{2}\left(\left\{l\right\}\right)=\alpha_{bayes,l},\forall l\in \{1,2,\ldots ,m\}\\ m_{1}\left(A\right)=0,\forall A\in 2^{\Theta }\setminus \{\{D_{1}\},\{D_{2}\},\ldots ,\{D_{m}\}\} \end{array}$$

Thirdly, the BPA functions composed of the original MDN evidence and the BET-derived evidence are combined using the DS combination rule (Equation (13)) to produce the final fused evidence:

(13) $$m_{1,2}\left(\left\{D_{i}\right\}\right)=m_{1}⨂m_{2}=\frac{1}{K}\sum_{B\cap C=A}m_{1}\left(B\right)\cdot m_{2}\left(C\right)$$

where K is:

(14) $$K=\sum_{B\cap C\neq \emptyset }m_{1}(B)\cdot m_{2}(C)$$

The final evidence after fusion is expressed as:

(15) $$m_{1,2}\left(\left\{D_{i}\right\}\right)=m^{*}\left(l\right)=\frac{m_{\mathrm{Bayes}}\left(l\right)\cdot \alpha_{l}}{\sum_{i=1}^{m}m_{\mathrm{Bayes}}\left(i\right)\cdot \alpha_{i}}$$

The component $l^{*}$ with the highest fused belief $m^{*}\left(l\right)$ is selected, and its corresponding mean ${u_{l}}^{*}$ is used as the next robot configuration point $c_{j+1}$:

(16) $$c_{j+1}=argmax\left(m_{1,2}\left(\left\{D_{i}\right\}\right)\right)={u_{l}}^{*}$$

Another parameter is passed into the next process.

(17) $$\sigma_{j+1}={\sigma_{l}}^{*}$$

The inputs and outputs process in the fusion sampling module can be simplified as follows:

(18) $$c_{j+1},\sigma_{j+1}\leftarrow {Sample}_{fused}(P\left(c_{j+1}|c_{j}\right))$$

The implementation of our fusion sampling method enhances traditional multinomial sampling by rigorously quantifying uncertainty and integrating multiple sources of evidence. Our fusion sampling module combines prior information and observation data averaged across components, thereby explicitly accounting for sampling uncertainty. The DS theory is used to merge the original MDN distribution with Bayesian estimates, effectively resolving conflicts between different sources favoring distinct Gaussian components. This evidence combination technique redistributes probability mass to minimize the impact of outliers and prediction errors. By independently applying fusion to each configuration dimension, our method accommodates the varying uncertainties across different robot joints. The implementation features vectorized operations for computational efficiency and numerical stability, with normalization ensuring valid probability distributions.

#### 3.2.3. Gaussian Sampling Module

The PSNet, through Gaussian resampling, can correct its collision errors. When collision points occur in the robot’s global configuration path generated by the FSM, these points, along with the subsequent point, are recorded as a collision set $c_{set}\in [{c_{j,}c_{j+1,}c}_{j+2}]$. This set is reconstructed as a multimodal Gaussian model using the provided parameters, and resampling is performed to correct the collision points within the path. The expression of the execution process of GSM can be simplified as:

(19) $${c_{j,\;}c_{j+1,\;}c}_{j+2}\leftarrow \;{Sample}_{Gaussian}(\left[{c_{j,\;}c_{j+1,\;}c}_{j+2}\right],\left[{\sigma_{j,\;}\sigma_{j+1,\;}\sigma }_{j+2}\right])$$

**Figure 2:**
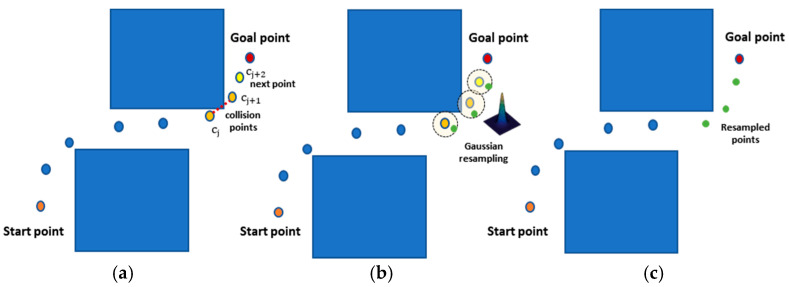
The schematic diagram of the Gaussian resampling module. (**a**) Record all collision points and the next point as a collision set; (**b**) Reconstruct multimodal Gaussian models and resample; (**c**) A repaired local path.

The schematic diagram is shown in Figure 2.

### 3.3. An Enhanced Artificial Potential Field

In most cases, the neural planner PSNet successfully computes a path solution. However, for some hard cases where the PSNet fails to find a path between beacon states, an enhanced artificial potential field (EAPF) planner is activated for replanning. The classical artificial potential field (CAPF) method is renowned for its efficiency and precision in local planning [47], but two significant drawbacks have limited its broader application [48,49]. The local minimum field traps the robot before reaching its global goal point. On the other hand, the repulsive field tends to repel the robot when it reaches a goal point close to an obstacle, which causes the goal to be a non-reachable phenomenon. To overcome these issues, a variable rotational field (VRF) is proposed to enhance the artificial potential field method to repair the local path when a failed case occurs in the global path planned by the PSNet.

The attractive and repulsive fields of CAPF can be computed by Equations (20) and (21), respectively.

(20) $$U_{at}=k_{at}d{\left(c_{init},c_{T}\right)}^{n_{at}}$$

where $U_{at}$ denotes the attractive field, and $k_{at}$ and $n_{at}$ are the gain coefficient and order of attractive functions, respectively.

(21) $$U_{re}=\left\{\begin{array}{c} k_{re}{\left(\frac{1}{d\left(c_{init},c_{T}\right)}-\frac{1}{d_{0}}\right)}^{n^{re}},\;if\;d\left(c_{init},c_{T}\right)\leq d_{0}\\ \;\;\;\;\;\;\;\;\;\;\;\;\;\;\;\;\;\;\;0\;\;\;\;\;\;\;\;\;\;\;\;\;\;\;\;\;\;\;\;\;\;\;\;,\;if\;d\left(c_{init},c_{T}\right)>d_{0} \end{array}\;\right.$$

where $U_{re}$ denotes the repulsive field, and $k_{re}$ and $n_{re}$ are the gain coefficient and order of repulsive functions, respectively.

Unlike the CAPF, the EAPF simplifies the repulsion field $U_{re}$ and introduces an extra variable field called the variable rotational field $U_{R}$. The EAPF is derived as follows.

The repulsive field of EAPF is simplified as:

(22) $$U_{re}=\underset{d\left(c_{\mathrm{init}},c_{T}\right)\to 0}{\lim}k_{re}{d\left(c_{init},c_{T}\right)}^{n^{re}}$$

Equation (23) means the repulsive influence of unobstructed areas is eliminated, so the goal non-reachable problem does not exist.

In addition, a new variable rotational field $U_{R}$ is defined as Equation (23) for solving the local minimum problem.

(23) $$U_{R}=f\left(x\right)S_{obs}\;f\left(x\right)=kx,k<0,x\in [1,lenth\left(S_{obs}\right)$$

where $S_{obs}$ is the obstacle’s perimeter, and k is the coefficient.

Thus, the total potential field can be denoted as:

(24) $$U_{total}={U_{at}+U}_{re}+U_{R}$$

**Figure 3:**
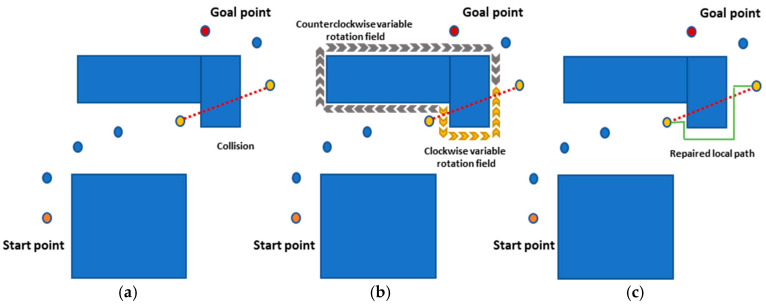
The schematic diagram of EAPF. (**a**) The planned global path falls into the worst scenario; (**b**) Create a variable rotational field with clockwise and counterclockwise directions, which is determined by the interior point method; (**c**) Repair the local path.

When the planned global path leads to a worst-case scenario, where PSNet alone cannot guarantee collision avoidance, as depicted in Figure 3a, the EAPF planner intervenes. EAPF uses Equation (22) to create a variable rotational field encompassing clockwise and counterclockwise directions (Figure 3b). The algorithm then evaluates the cost function based on the shortest distance and selects the corresponding rotational field direction. This selected rotational field is added to the total potential field $U_{total}$. A viable path solution is ultimately generated by applying a gradient optimizer within this modified potential field (Figure 3c).

The expression of the execution process of EAPF can be simplified as follows:

(25) $$c_{j+1}\leftarrow \;EAPF(c_{j},c_{j+1})$$

### 3.4. PSNet- EAPF

The previous section provides a detailed description of each component of the proposed method. Algorithm 1 outlines the complete execution process of the proposed PSNet-EAPF method for robotic motion planning in semi-structured environments. The algorithm takes as input the robot’s initial configuration $c_{init}$, goal configuration $c_{T}$, and environment information $O_{obs}$ representing obstacles. The algorithm first initializes the path k with just the start configuration $c_{init}$ (line 1). Then it enters a loop that continues to recursively call PSNet until the termination condition is satisfied, which is either finding a valid path to the goal or reaching a maximum iteration threshold (lines 2–23).

Inside the loop, the MPM of PSNet takes the current configuration $c_{j}$, goal $c_{T}$, and encoded environment information z to generate multimodal distribution parameters for the next configuration $c_{j+1}$ (lines 4 and 5). If the sampled $c_{j+1}$ is collision-free (lines 6 and 7), it is added to the path k (line 9). Otherwise, the Gaussian resampling function is invoked to resample the collision set $\left[{c_{j,}c_{j+1,}c}_{j+2}\right]$ using the provided distribution parameters to try to resolve the collision (lines 12–14).

If PSNet fails to resolve the collision, the EAPF method is used to replan a valid local path segment from $c_{j}$ to a new $c_{j+1}$ that avoids obstacles (line 21). The process repeats with the new $c_{j+1}$ until the goal $c_{T}$ is reached within the iteration limit, returning the planned collision-free path k (line 23), or the iteration limit is exceeded, returning failure. The PSNet-EAPF enhances the planner’s ability to produce high-quality trajectories by hybrid leveraging both learned experience and classical planning techniques.
**Algorithm 1: PSNet-EAPF****Input:** environmental information $O_{obs}$, initial state $c_{init},\;$ goal state $c_{T}$
**Output:** Collision-free path solution $Patch\;k=\{c_{init},c_{2},\ldots ,c_{T}\}$
1: 2: 3: 4: 5: 6: 7: 8: 9: 10: 11: 12: 13: 14: 15: 16: 17: 18: 19: 20: 21: 22: 23:
Initialize Φ($c_{j},c_{j+1},O_{obs}$)*,*$\;Patch\;k\leftarrow [c_{init}]$ **while ***Termination Condition Unsatified ***do** $z\leftarrow Enc(O_{obs})$; $P(c_{j+1}|c_{j})\leftarrow PSNet(\{c_{j},c_{j+1},z\})$; $c_{j+1},\sigma_{j+1}\leftarrow {Sample}_{fused}(P(c_{j+1}|c_{j}))$; Check Motion: $\{valid,collision\}\leftarrow \Phi (c_{j},c_{j+1},O_{obs}$); **If **valid**then**
 $Patch\;k.append\left(c_{j+1}\right)$; **else**  **for**
j=1* to Iterations Number*  **do**    $P(c_{j+2}\;|\;c_{j+1})\leftarrow PSNet(\{c_{j+1},c_{T},z\})$;   $\;c_{j+2},\sigma_{j+2}\leftarrow {Sample}_{fused}(P(c_{j+2}|c_{j+1}))$;     $c_{j},c_{j+1},c_{j+2}\leftarrow {Sample}_{Gaussian}([c_{j},c_{j+1},c_{j+2}],[\sigma_{j},\sigma_{j+1},\sigma_{j+2}])$;    Check Motion:    $\{valid,collision\}\leftarrow \Phi ([c_{j},c_{j+1},c_{j+2}],O_{obs}$);  **If**
valid
**then**    $Patch\;k\left[j:j_{+2}\right]=c_{j},c_{j+1},c_{j+2}$;
    **break;**
  **If**
collision
**then**    $c_{j+1}\leftarrow \;EAPF(c_{j},c_{j+1})$;    $Patch\;k.append\left(c_{j+1}\right)$ **return**
Patch k;

## 4. Experiments

In this section, the effectiveness of the proposed PSNet-EAPF method is evaluated through comparative analysis against two established planners: MPNet, a state-of-the-art network planner that has proven its worth over numerous sampling-based optimization techniques in terms of execution time, and RRT-connect, a classical planner renowned for its efficiency. For the evaluation, two distinct planning challenges have been selected: one involving a 2-D point-mass robot and another encompassing a 6-degree-of-freedom (DOF) universal robot manipulator. These diverse scenarios serve to underscore the versatility and robustness of the PSNet-EAPF approach across a range of robotic platforms and environments. The system used for experiments has an Intel Core i5 processor with 32-GB RAM and a GeForce GTX 2060 GPU. A detailed specification is presented in Table 1.

Considering that implementation details and engineering optimizations may interfere with the measurement of execution time, this paper uses the iteration count as a stable efficiency metric in the overall evaluation. In the high-dimensional (6-DOF) multi-joint robot experiments, however, since the time consumption of different methods differs by several orders of magnitude, the execution time is additionally reported to directly reflect the performance difference, even though no efficiency optimization has been applied to our code framework.

### 4.1. 2-D Point-Mass Robot

In planning a 2-D point-mass robot, three workspaces with varying complexity levels (simple, mid-complex, and complex) containing 2280 trajectories were used to train both PSNet-EAPF and MPNet. The trained models were then evaluated on separate test sample sets, each with thirty new and unseen start and goal configurations per scenario. Additionally, evaluation tests were conducted multiple times on the test sample sets for all methods compared in our experiments, and the mean values of their results were reported.

**Figure 4:**
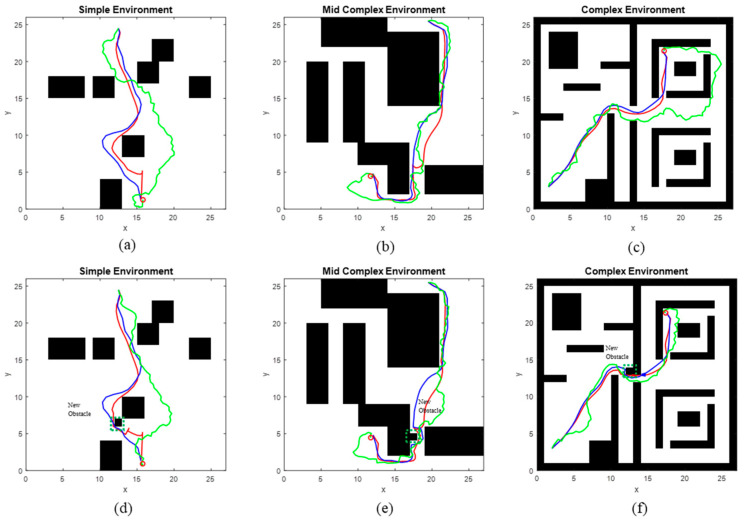
Path comparison of PSNet-EAPF (red) with MPNet (blue) and RRT-connect (green) in example environments. (**a**–**c**) represent Simple 2-D, Mid-complex 2-D, and Complex 2-D environments, respectively; (**d**–**f**) represent the addition of new obstacles (The area marked with green dashed lines) in Simple 2-D, Mid-complex 2-D, and Complex 2-D environments, respectively.

Figure 4 illustrates the paths planned by PSNet-EAPF (red), its competitor MPNet (blue), and RRT-connect (green). It can be observed that PSNet-EAPF planned paths similar to MPNet and outperformed RRT-connect in terms of robustness and shorter path lengths.

**Figure 5:**
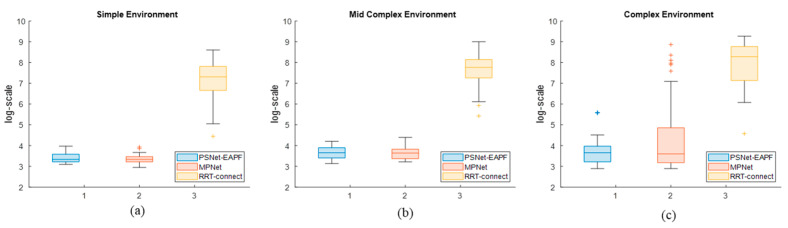
The average iteration number distribution in log-scale of the planners in different environments. The box plots exhibit the interquartile range, minimum, and maximum iteration number distribution of PSNet-EAPF, MPNet, and RRT-connect in the Simple environment (**a**), Mid-complex environment (**b**), and Complex environment (**c**).

Figure 5 shows that the average number and dispersion of iterations in log-scale for PSNet-EAPF and MPNet are significantly better than for RRT-connect across all test environments. While PSNet-EAPF and MPNet exhibit similar performance in the simple environment, MPNet’s performance progressively worsens with increasing environmental complexity. This decline is most pronounced in the complex environment, where MPNet suffers a dramatic loss in both iteration efficiency and stability.

**Figure 6:**
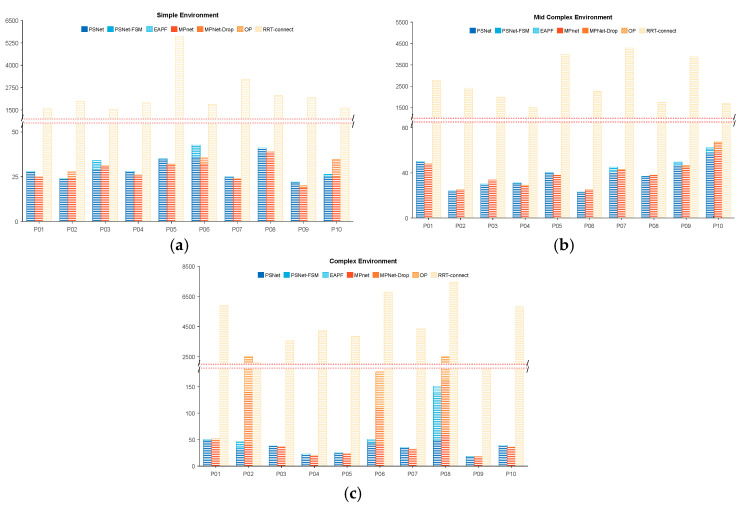
The iteration number and percentage of each module of planners in different environments. (**a**) Statistics of the iteration counts and proportions of different method modules in a simple environment; (**b**) Statistics of the iteration counts and proportions of different method modules in a mid-complex environment; and (**c**) Statistics of the iteration counts and proportions of different method modules in a complex environment.

Figure 6 provides a detailed analysis of the percentage of iteration cost associated with ten randomly sampled beacon pairs from thirty new and unseen start and goal configurations for each module of the evaluated methods. Across all tested samples, PSNet and the equivalent MPNet module within MPNet-Oracle both consistently require 20–50 iterations for global path generation, showing no statistically significant difference in base planning capability. If collisions occur in the planned global path, the path repair functions of Gaussian resampling (PSNet-FSM) and the dropout layer (MPNet-Drop), integrated within PSNet and MPNet, respectively, are activated to address these local collision paths. MPNet-Drop has a lower repair success rate than PSNet-FSM and frequently fails in worst-case scenarios. Particularly in complex scenarios, as shown in Figure 6c, the former has a 30% probability of failure, while the latter has only 10%. In such cases, the MPNet requires invoking an additional Oracle planner (OP), a sampling algorithm that consumes additional computational resources. In contrast, PSNet-FSM can efficiently help PSNet repair these collision paths. Even in extreme cases like certain collision points shown in the P08 group in Figure 6c, EAPF can quickly find a collision-free path through dozens of iterations, avoiding the need for thousands of iterations required by sampling algorithms.

**Figure 7:**
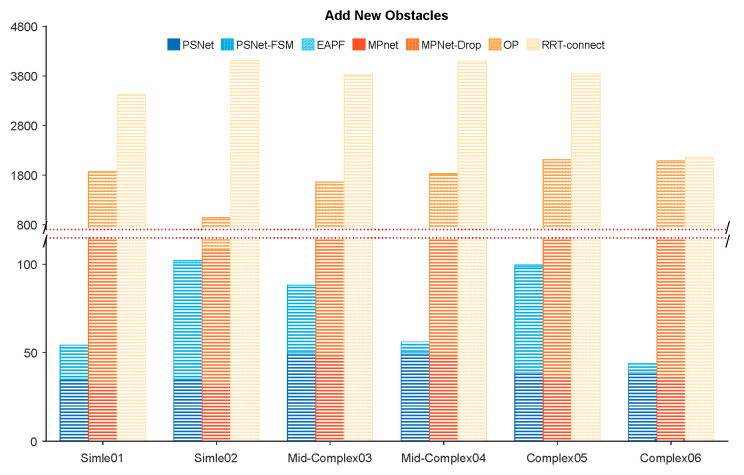
The iteration number and percentage of each module of planners in different environments (Add new obstacles).

Furthermore, a new obstacle was temporarily added to each test environment to evaluate the algorithms’ ability to handle unexpected changes, specifically designed to disrupt the planned global paths (Figure 4d–f). We have still randomly chosen some sets of results from all experimental groups, as shown in Figure 7. The results show that PSNet can rely on its structure (PSNet-FSM) to avoid almost all temporary obstacle blockages within 50–100 iterations. In contrast, MPNet performs poorly when using its dropout structure to find obstacle-free paths, failing to avoid obstacles in almost all samples even after 100 dropout operations, and instead requiring the invocation of the Op for 800–1800 iterations of computation. Its computational cost in this environment, as tested, is significantly higher than our method, similar to RRT-connect.

### 4.2. 6-DOF Universal Robot Manipulator

**Figure 8:**
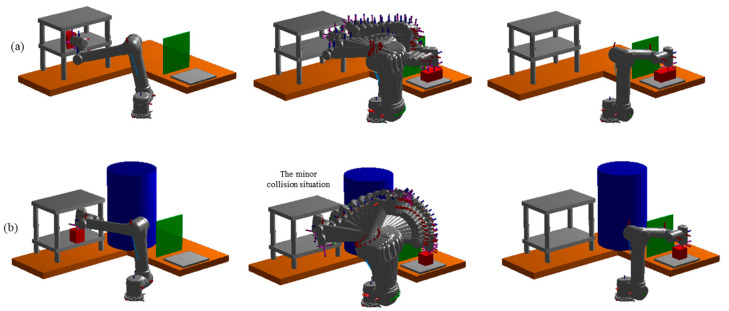
The robot pick-and-place task environment. The two test environment settings. Test environment two (**b**) added more obstacles than test environment one (**a**) and required the robot manipulator to reach into the grey storage rack, a narrow space, to pick up the red workpiece.

In the high-dimensional motion planning problem for a 6-DOF universal robot manipulator, the planning task is formulated as finding collision-free joint-space trajectories (i.e., sequences of 6-dimensional joint angle configurations) from an initial configuration to a goal configuration in the configuration space ($C\subset R^{6}$). The goal configurations are defined such that the end-effector can successfully grasp the red workpiece from the gray storage rack and place it onto the operation platform. The planner generates a trajectory $k=\{c_{init},c_{2},\ldots ,c_{T}\}$, where each $c_{j}$ represents a 6-DOF joint angle vector. The robot training dataset contains three industrial environments for pick-and-place tasks with two hundred trajectories each. The robot test dataset includes the same two work environments as in training but with thirty new and unseen start and goal configurations per scenario. The evaluation tests were conducted five times on the test sample sets for all methods compared in our experiments, and the mean values of their results were reported. The planning task environment contains a brown ‘L’ shaped workbench with a grey storage rack, a green protector plate, a grey operation platform, and a blue pillar randomly added and placed within the environment. The planner’s job is to find a collision-free path for the robot to pick up a red workpiece from the grey storage rack and place it on the operation platform while avoiding environmental obstacles. Figure 8 illustrates the two test environment settings. The first test environment (Figure 8a) presents a moderately cluttered scenario, while the second test environment (Figure 8b) introduces a higher degree of complexity by incorporating additional obstacles. In addition, the robot manipulator must navigate through a constricted space within the grey storage rack to successfully grasp and retrieve the red workpiece in the second environment.

**Figure 9:**
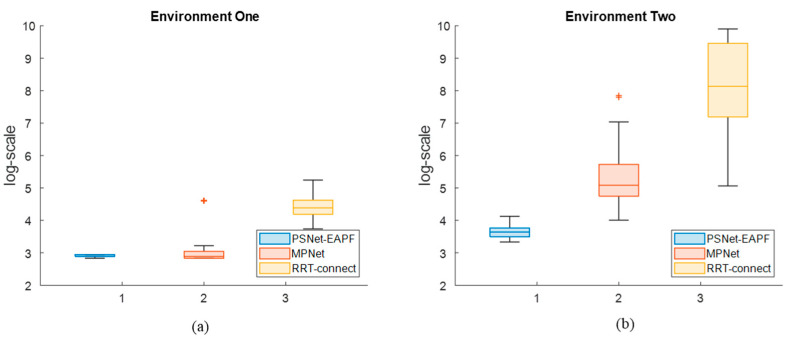
The interquartile results of iterations, in log-scale, over the entire 6-DOF robot test dataset. The plot illustrates the median, interquartile range (IQR), minimum, and maximum number of iterations required by PSNet-EAPF, MPNet, and RRT-Connect across all test environments. (**a**) The test values in environment one; (**b**) The test values in environment two.

Figure 9 illustrates the interquartile results of iterations, in log-scale, over the entire 6-DOF robot test dataset. The proposed method maintains lower median levels than other competitors, with values of 2.89 and 3.638, and interquartile ranges (IQR) of approximately 0.054 and 0.264, respectively. MPNet-Oracle exhibits sensitivity to changes in the planning environment. Its median and IQR in environment one are comparable to ours but significantly higher in environment two, increasing from 2.89 (IQR 0.212) to 5.084 (IQR 3.028). In all test environments, the medians and IQR of RRT-connect are higher than those of our method and MPNet-Oracle. These values increase exponentially as the planning environment degrades.

**Figure 10:**
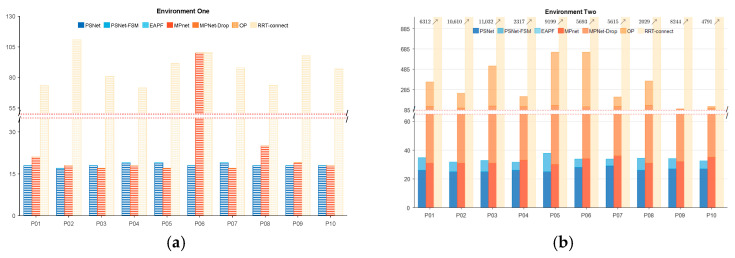
The iteration number and percentage of each module of planners in different environments. The blue, orange, and yellow bars indicate the PSNet, MPnet, and RRT-connect groups. (**a**) Statistics of the iteration counts and proportions of different method modules in the environment one environment and (**b**) Statistics of the iteration counts and proportions of different method modules in the environment two.

We analyzed the computational overhead associated with each method’s modules for a more detailed evaluation of the proposed PSNet-EAPF algorithm and its competitors, MPNet and RRT-Connect. Figure 10 illustrates the percentage of iteration cost attributed to each module of the evaluated methods for ten sets of randomly sampled beacon pairs. In environment one, PSNet-EAPF, on average, finds global paths in 20 iterations by invoking only the MF module. MPNet-Oracle also found global paths with similar iterations as PSNet-EAPF, except for sample P06, but its collision avoidance ability was weak. MPNet-Oracle struggled to rely on its drop module to repair local paths and avoid collisions. MPNet-Oracle appears to struggle in slightly complex environments when relying on its drop module to repair local paths and avoid obstacles, as only one out of 10 tests in Figure 10b successfully used the drop module to resolve collision paths. Even minor ones that brush against the edge of the shelf in Figure 8b require additional OP for resampling planning, resulting in higher computational overhead and reduced consistency.

In the multi-joint robot experiments, this study compared the iterative test results of various method modules while recording their computation time and corresponding standard deviations (SD). As shown in Table 2, in the relatively simple obstacle environment (environment one), both PSNet-EAPF and MPNet-Oracle were able to complete path planning mainly through the network planner within approximately 0.3 s (only MPNet-Oracle invoked the Drop module in a few test cases). Although MPNet-Oracle demonstrated comparable performance in simple environments, its time cost and variance increased significantly in complex dynamic scenarios due to its dependence on the computationally expensive OP. RRT-Connect, as a pure sampling-based method, exhibits the highest time consumption, often exceeding 6 s (≈21.6 × faster), and shows the largest standard deviation, indicating unpredictable performance.

Table 3 reports the planning time of each method in environment two. In this more complex setting, the advantage of PSNet-EAPF is even more pronounced, with an average planning time of only 5.354 s and a standard deviation of 1.137 s. By contrast, MPNet-Oracle’s average computation time soars to 228.818 s (≈42.7 × faster), accompanied by a large std 203.714 s (≈55.3 × faster). This dramatic increase arises because the Drop module in MPNet struggles in high-dimensional, complex environments, prompting frequent calls to the computationally expensive Oracle Planner. As a result, the planning process transitions from rapid, network-based inference to slow random sampling and collision detection, escalating the runtime from seconds to minutes. At the same time, RRT-Connect continues to exhibit the highest computational burden among all methods.

**Figure 11:**
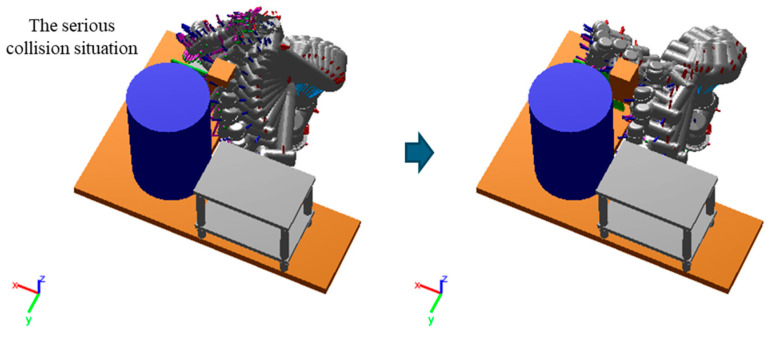
An unknown obstacle is added to the necessary road of the robot manipulator to disrupt the continuous global path.

**Figure 12:**
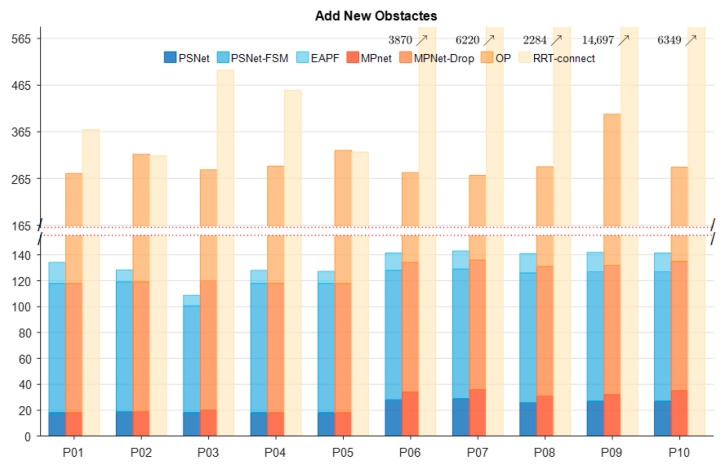
The iteration number and percentage of each module of planners in different environments (add the new obstacle).

To further evaluate each method’s capability to escape collisions, an unknown obstacle was added into the robot manipulator’s path, obstructing the continuous global path, as shown in Figure 11. This scenario created a serious collision situation, testing the algorithms’ ability to react and replan in unexpected obstacles. The statistical results of this experiment, involving five start and goal pair configurations in both the first (P01–P05) and the more challenging second (P06–P10) test environments, were presented in Figure 12. While both MPNet-Oracle and PSNet-EAPF face difficulties in handling such critical collision scenarios, the PSNet-EAPF method leverages the EAPF to efficiently identify near-optimal collision-free paths (Only a few dozen iterations are needed.), avoiding the disorderly search behavior exhibited by sampling-based algorithms. In contrast, the MPNet-Oracle and RRT-Connect algorithms exhibit high computational costs and significant performance variability across similar planning problems. (It requires hundreds or even tens of thousands of iterative calculations.) This highlights the effectiveness of EAPF in enhancing collision avoidance and path replanning capabilities, particularly in complex environments with unexpected obstacles.

Table 4 further illustrates the runtime of different methods in an experimental environment with newly added obstacles. The average computation time of PSNet-EAPF increases from 0.304 s and 5.354 s in environments one and two, respectively, to 11.284 s, with a corresponding rise in standard deviation. Nevertheless, compared with MPNet-Oracle and RRT-Connect, whose average runtimes reach approximately 300 s (≈30.6× and ≈26.9× faster) with standard deviations exceeding 200 s, PSNet-EAPF still demonstrates a distinct advantage.

Based on the results of the above experiments, we realized that network accuracy issues became more pronounced in high-dimensional motion planning, potentially causing oscillations in the global path near the target point. These oscillations could lead to planning failures and increase the likelihood of encountering worst-case scenarios. To quantify the robustness of the evaluated methods in addressing this challenge, Table 5 counted the global planning success rates achieved by each algorithm across all test tasks. PSNet showed a much higher success rate than MPNet in planning collision-free paths to new target points in the original environment. This suggests that PSNet effectively addresses most minor collision issues stemming from network inaccuracy without encountering the worst-case scenarios that can trap MPNet due to its lack of necessary randomness from dropout. However, for severe collisions caused by new unknown obstacles that appear to surpass the level of network inaccuracy-induced collisions, both PSNet and MPNet exhibit unsatisfactory performance, necessitating the intervention of their respective local planners for correction. This discrepancy in performance highlights the effectiveness of PSNet in mitigating the adverse effects of network accuracy errors on global planning, thereby enhancing the overall reliability and robustness of the motion planning system in high-dimensional scenarios.

## 5. Discussion

### 5.1. Neural Network-Based Global Planner-PSNet

MPNet stands as a distinguished neural network-based planning approach, renowned for its adeptness at tackling intricate planning conundrums with remarkable efficiency. Nonetheless, it grapples with network accuracy constraints, resulting in local collisions and oscillations in the vicinity of the target point—hindrances that undermine its capacity to evade collisions effectively. Our method circumvents these shortcomings by revamping the network output layer architecture to engender multiple multimodal distributions and refining the final path via a sophisticated data fusion module. Concretely, the output layer has been meticulously engineered to spawn an array of multimodal distributions, which are subsequently parsed and amalgamated through the data fusion module. This transformative enhancement endeavors to curtail the ramifications of network accuracy discrepancies on the crafted trajectory and facilitates the development of Gaussian resampling capabilities. PSNet can solve global paths for all scenarios in the experiment within almost 50 iterations, without requiring hundreds or even thousands of iterative calculations like sampling algorithms.

In essence, the proposed PSNet method pays homage to the robust attributes of classical neural network-based planning paradigms in expeditiously calculating global paths, all the while alleviating their inherent limitations through strategic architectural refinements.

### 5.2. Local Replanning-EAPF

Local replanning plays a pivotal role in the proposed motion planning method, as network accuracy errors during global path computation may result in local collisions along the planned global trajectory. The MPNet algorithm attempts to address these local collision issues through a stochastic approach, employing active drop layers that randomly mask neurons, consequently introducing different probability distributions in each planning step. However, this approach often fails to provide satisfactory solutions, particularly in high-dimensional collision scenarios, as the converged network offers limited randomness for effective replanning. So, an additional BNP is required for resampling planning, resulting in higher computational overhead and reduced consistency. PSNet-FSM successfully resolves 89.4% of collision scenarios in 2-D tasks and 87.8% in 6-DOF tasks within 15–95 iterations. In contrast, MPNet-Drop exhibits a 32.9% failure rate in original test environments and a 68.1% failure rate when unexpected obstacles are introduced.

When improving our network planner, we considered equipping PSNet with Gaussian sampling capabilities, allowing it to address collision points correctly while planning the global path promptly, thereby avoiding the abovementioned issues. If PSNet’s global path encounters worst-case scenarios, the local replanning method EAPF, which enhances the variable rotational field to overcome the limitations of local minima and the repulsive field found in traditional potential field approaches, is activated. The EAPF’s capability to resolve worst-case scenarios in dozens of iterations instead of thousands is a primary contributor to the stable and low time cost observed in complex environments (Section 4.2).

## 6. Conclusions

This study presents a pioneering approach that synergistically combines a probabilistic sampling network with an enhanced artificial potential field to efficiently resolve intricate planning challenges within semi-structured environments. By incorporating the innovative IMDN model into the conventional motion planning network, a multitude of Gaussian distributions are generated, effectively mitigating network accuracy errors. Furthermore, we embed a Gaussian resampling function within our PSNet framework to bolster its local collision evasion prowess. Complementing these advancements, an enhanced artificial potential field, addressing local minima and repulsive field limitations, is included as an independent module to handle worst-case scenarios. The experimental results provide quantitative validation of our method’s advantages. PSNet-EAPF achieved a reduction in median iteration count by an order of magnitude, from thousands to tens or low hundreds, in high-complexity environments. This iteration efficiency directly translated into a drastic reduction in wall-clock planning time. Our method consistently generated paths in seconds (e.g., 5.354 s in a complex 6-DOF task), while baseline methods frequently required minute-scale times (e.g., 228.818/295.849 s for MPNet-Oracle) under the same conditions.

Future work will focus on refining the algorithm and extending its applicability to broader robotic platforms. The current design primarily emphasizes functional realization, leaving substantial room for improving computational efficiency. Therefore, improving the overall algorithm performance by optimizing redundant results in modules such as Gaussian sampling and refining the code framework will become the core objective of the next phase. Although the current validation has been conducted on a high-degree-of-freedom manipulator system, the proposed algorithmic framework possesses inherent transferability to other robotic platforms. We plan to explore its application in mobile robot navigation and investigate cooperative planning problems in mobile manipulator systems, particularly addressing the complex challenges arising from coordinating chassis motion with arm configuration.

## Figures and Tables

**Table 1:** Hardware and Software Versions for the Experiment.

Main Hardware	Software Platform
I5-10400 CPU	Matlab R2021b
RTX2060 GPU	Python 3.7.7

**Table 2:** Time cost of methods in environment one.

Method	PSNet-EAPF		MPNet-Oracle		RRT-Connect	
	Time (s)	SD (s)	Time (s)	SD (s)	Time (s)	SD (s)
PSNet/MPNet	0.304	0.020	0.303	0.023	/	/
PSNet-FSM/MPNet-Drop	0.000	0.000	0.016	0.048	/	/
EAPF/Oracle	0.000	0.000	0.000	0.000	/	/
Complete operation	0.304	0.020	0.319	0.065	6.558	12.561

**Table 3:** Time cost of methods in environment two.

Method	PSNet-EAPF		MPNet-Oracle		RRT-Connect	
	Time (s)	SD (s)	Time (s)	SD (s)	Time (s)	SD (s)
PSNet/MPNet	0.703	0.616	0.957	0.736	/	/
PSNet-FSM/MPNet-Drop	4.633	0.890	20.822	3.354	/	/
EAPF/Oracle	0.018	0.099	207.039	203.719	/	/
Complete operation	5.354	1.137	228.818	203.714	295.849	225.811

**Table 4:** Add new obstacles Method’s time loss.

Method	PSNet-EAPF		MPNet-Oracle		RRT-Connect	
	Time (s)	SD (s)	Time (s)	SD (s)	Time (s)	SD (s)
PSNet/MPNet	0.735	0.658	0.954	0.746	/	/
PSNet-FSM/MPNet-Drop	10.028	0.895	63.164	4.164	/	/
EAPF/Oracle	0.520	0.176	280.718	204.257	/	/
Complete operation	11.284	1.104	344.836	204.219	303.402	292.842

**Table 5:** Method’s global planning success rate across all test tasks.

Method	The Success Rate of the Global Path	
	Original Environments	Added Unknown Obstacle
MPNet	66.3%	32.9%
PSNet	100.0%	51.1%

## Data Availability

Data will be made available upon request.

## References

[1] Liu Q., Liu Z., Xu W., Tang Q., Zhou Z., Pham D.T. (2019). Human-robot collaboration in disassembly for sustainable manufacturing. Int. J. Prod. Res..

[2] Liu W., Liu C., Liang X., Zheng M. (2024). A hybrid task-constrained motion planning for collaborative robots in intelligent remanufacturing. Mechatronics.

[3] De Pace F., Manuri F., Sanna A., Fornaro C. (2020). A systematic review of Augmented Reality interfaces for collaborative industrial robots. Comput. Ind. Eng..

[4] LaValle S.M., Kuffner J.J. (2001). Rapidly-Exploring Random Trees: Progress and Prospects. Algorithmic and Computational Robotics.

[5] Wu Z., Meng Z., Zhao W., Wu Z. (2021). Fast-RRT: A RRT-Based Optimal Path Finding Method. Appl. Sci..

[6] Zhuge C., Wang Q., Liu J., Yao L. (2021). An Improved Q-RRT* Algorithm Based on Virtual Light. Comput. Syst. Sci. Eng..

[7] Li Y., Wei W., Gao Y., Wang D., Fan Z. (2020). PQ-RRT*: An improved path planning algorithm for mobile robots. Expert Syst. Appl..

[8] Qiu Q., Cao Q. (2020). Motion planning in semi-structured environments with teaching roadmaps. Intel. Serv. Robot..

[9] Liu H., Dong W., Zhang Z., Wang C., Li R., Gao Y. (2024). Optimization-based local planner for a nonholonomic autonomous mobile robot in semi-structured environments. Robot. Auton. Syst..

[10] Lin H.-I. (2020). Learning on robot skills: Motion adjustment and smooth concatenation of motion blocks. Eng. Appl. Artif. Intel..

[11] Jin T., Kobayashi T., Matsubara T. (2024). Constrained footstep planning using model-based reinforcement learning in virtual constraint-based walking. Adv. Robot..

[12] Burns B., Brock O. (2005). Sampling-based motion planning using predictive models. Proceedings of the 2005 IEEE International Conference on Robotics and Automation.

[13] Kicki P., Gawron T., Ćwian K., Ozay M., Skrzypczyński P. (2021). Learning from experience for rapid generation of local car maneuvers. Eng. Appl. Artif. Intel..

[14] Lyu E., Liu T., Wang J., Song S., Meng M.Q.-H. (2022). Motion Planning of Manipulator by Points-Guided Sampling Network. IEEE Trans. Autom. Sci. Eng..

[15] Jetchev N., Toussaint M. (2013). Fast motion planning from experience: Trajectory prediction for speeding up movement generation. Auton Robot..

[16] Wang J., Zhang T., Ma N., Li Z., Ma H., Meng F., Meng M.Q. (2021). A survey of learning based robot motion planning. IET Cyber-Syst. Robot..

[17] Qureshi A.H., Miao Y., Simeonov A., Yip M.C. (2020). Motion planning networks: Bridging the gap between learning-based and classical motion planners. IEEE Trans. Robot..

[18] Lozano-Pérez T., Wesley M.A. (1979). An algorithm for planning collision-free paths among polyhedral obstacles. Commun. ACM.

[19] Schweikard A. (1992). A simple path search strategy based on calculation of free sections of motions. Eng. Appl. Artif. Intell..

[20] Latombe L.E.K.J.-C. (1998). Probabilistic roadmaps for robot path planning. Pratical Motion Planning in Robotics: Current Aproaches and Future Challenges.

[21] Palmieri G., Scoccia C. (2021). Motion planning and control of redundant manipulators for dynamical obstacle avoidance. Machines.

[22] Kuffner J.J., LaValle S.M. (2020). RRT-connect: An efficient approach to single-query path planning. Proceedings of the 2000 ICRA. Millennium Conference, IEEE International Conference on Robotics and Automation, Symposia Proceedings (Cat. No. 00CH37065).

[23] Wang K., Huang B., Zeng G., Li X.B. (2019). Faster path planning based on improved RRT-connect algorithm. J. Wuhan Univ. (Nat. Sci. Ed.).

[24] Chen J., Zhao Y., Xu X. (2021). Improved RRT-Connect Based Path Planning Algorithm for Mobile Robots. IEEE Access.

[25] Karaman S., Frazzoli E. (2011). Sampling-based algorithms for optimal motion planning. Ind. Robot..

[26] Nasir J., Islam F., Malik U., Ayaz Y., Hasan O., Khan M., Muhammad M.S. (2013). RRT*-SMART: A rapid convergence implementation of RRT. Int. J. Adv. Robot. Syst..

[27] Fan J., Chen X., Wang Y., Chen X. (2022). UAV trajectory planning in cluttered environments based on PF-RRT* algorithm with goal-biased strategy. Eng. Appl. Artif. Intel..

[28] Ichter B., Harrison J., Pavone M. (2018). Learning sampling distributions for robot motion planning. Proceedings of the 2018 IEEE International Conference on Robotics and Automation (ICRA).

[29] Wang Y., Harada K., Wan W. (2020). Motion planning of skillful motions in assembly process through human demonstration. Adv. Robot..

[30] Fisher R., Rosman B., Ivan V. (2018). Real-time motion planning in changing environments using topology-based encoding of past knowledge. Proceedings of the 2018 IEEE/RSJ International Conference on Intelligent Robots and Systems (IROS).

[31] Zuo G., Wu C., Huang G. (2023). Repetitive Path Planning with Experience-Based Bidirectional RRT. International Conference on Robots for Space Applications in Orbital Stations.

[32] Roveda L., Magni M., Cantoni M., Piga D., Bucca G. (2021). Human–robot collaboration in sensorless assembly task learning enhanced by uncertainties adaptation via Bayesian Optimization. Robot. Auton. Syst..

[33] Zuo G., Li M., Yu J., Wu C., Huang G. (2022). An efficient motion planning method with a lazy demonstration graph for repetitive pick-and-place. Biomimetics.

[34] Hartmann V.N., Ortiz-Haro J., Toussaint M. (2023). Efficient path planning in manipulation planning problems by actively reusing validation effort. Proceedings of the 2023 IEEE/RSJ International Conference on Intelligent Robots and Systems (IROS).

[35] Ichter B., Pavone M. (2019). Robot motion planning in learned latent spaces. IEEE Robot. Autom. Lett..

[36] Wang J., Chi W., Li C., Wang C., Meng M.Q.-H. (2020). Neural RRT*: Learning-based optimal path planning. IEEE Trans. Autom. Sci. Eng..

[37] Wang H., Li Y. (2024). Limited environmental information path planning based on 3D point cloud reconstruction. J. Supercomput..

[38] Nguyen H., Andersen R., Boukas E., Alexis K. (2024). Uncertainty-aware visually-attentive navigation using deep neural networks. Int. J. Robot. Res..

[39] Zhou B., Yi J., Zhang X., Chen L., Yang D., Han F., Zhang H. (2022). An autonomous navigation approach for unmanned vehicle in outdoor unstructured terrain with dynamic and negative obstacles. Robotica.

[40] Janson L., Schmerling E., Clark A., Pavone M. (2015). Fast marching tree: A fast marching sampling-based method for optimal motion planning in many dimensions. Int. J. Robot. Res..

[41] Li H., Wang Y., Guo Y., Duan J. (2025). Vole foraging-inspired dynamic path planning of wheeled humanoid robots under workshop slippery road conditions. Biomimetics.

[42] Qureshi A.H., Dong J., Choe A., Yip M.C. (2020). Neural manipulation planning on constraint manifolds. IEEE Robot. Autom. Lett..

[43] Ying K.-C., Pourhejazy P., Cheng C.-Y., Cai Z.-Y. (2021). Deep learning-based optimization for motion planning of dual-arm assembly robots. Comput. Ind. Eng..

[44] Li L., Miao Y., Qureshi A.H., Yip M.C. (2021). MPC-MPNet: Model-predictive motion planning networks for fast, near-optimal planning under kinodynamic constraints. IEEE Robot. Autom. Lett..

[45] Zhang H., Heiden E., Nikolaidis S., Lim J.J., Sukhatme G.S. (2018). Auto-conditioned recurrent mixture density networks for learning generalizable robot skills. arXiv.

[46] Ji X., Ren Y., Tang H., Shi C., Xiang J. (2020). An intelligent fault diagnosis approach based on Dempster-Shafer theory for hydraulic valves. Measurement.

[47] Choi D., Chhabra A., Kim D. (2022). Intelligent cooperative collision avoidance via fuzzy potential fields. Robotica.

[48] Song J., Hao C., Su J. (2020). Path planning for unmanned surface vehicle based on predictive artificial potential field. Int. J. Adv. Robot. Syst..

[49] Wang C., Chen D., Liao W., Liang Z. (2022). Autonomous obstacle avoidance strategies in the mission of large space debris removal using potential function. Adv. Space Res..

